# Health Literacy is a Rising Star in Policy and Research, also in Slovenia

**DOI:** 10.2478/sjph-2026-0008

**Published:** 2026-06-01

**Authors:** Mitja Vrdelja, Sanja Vrbovšek, Kristine Sørensen

**Affiliations:** National Institute of Public Health, Trubarjeva cesta 2, 1000 Ljubljana, Slovenia; Global Health Literacy Academy & Aalborg University, Borresøvej 26, 8240 Risskov, Denmark

**Keywords:** Health literacy, Organisational health literacy, Policy and research, Slovenian National Health Literacy Strategy, zdravstvena pismenost, organizacijska zdravstvena pismenost, politika in raziskave, slovenska nacionalna strategija zdravstvene pismenosti

## Abstract

The World Health Organization has identified health literacy as a key pillar for resilient health systems in its current global strategy for 2025–2028. In this editorial, we argue that effectively addressing health literacy requires its integration into key strategic frameworks at both the global and national levels, as this represents a fundamental precondition for a more coordinated and systematic approach to the issue. Slovenia has followed these global directions by adopting the National Health Literacy Strategy 2025–2035 in 2025, establishing a ten-year strategic framework to strengthen health literacy. The country is also adhering to recommendations for ongoing research in this field; in 2026, the second national health literacy survey will be conducted. Looking ahead, the focus should be on developing and implementing practical public health interventions, and on strengthening coordination with existing health promotion and prevention programmes in Slovenia that are already delivering measurable impact. A key challenge will be to strengthen collaboration between researchers, policy-makers, and practitioners to help create a supportive, health-literate environment in Slovenia.

## Izvleček

Svetovna zdravstvena organizacija je v svoji aktualni globalni strategiji za obdobje 2025—2028 poudarila, da je zdravstvena pismenost eden ključnih stebrov odpornosti zdravstvenih sistemov. V tem uvodniku zato razpravljamo, da je za učinkovito naslavljanje zdravstvene pismenosti nujno njeno umeščanje v ključne strateške dokumente tako na globalni kot nacionalni ravni, saj to predstavlja temeljni pogoj za bolj usklajeno in sistematično obravnavo tega področja. Slovenija tem globalnim usmeritvam sledi, saj je leta 2025 sprejela Nacionalno strategijo zdravstvene pismenosti 2025—2035, s čimer je vzpostavila dolgoročni strateški okvir za razvoj in krepitev zdravstvene pismenosti. Hkrati sledimo usmeritvam glede kontinuiranega raziskovanja na tem področju – v letu 2026 poteka druga nacionalna raziskava zdravstvene pismenosti, ki bo omogočila vpogled v trenutno stanje in spremljanje trendov skozi čas. V prihodnje bo pomemben poudarek na razvoju in implementaciji konkretnih javnozdravstvenih intervencij ter na boljši koordinaciji z že obstoječimi promocijskimi in preventivnimi programi v Sloveniji, ki izkazujejo pomembne javnozdravstvene učinke. Ključni izziv bo predvsem krepitev povezovanja med raziskovalci, odločevalci in strokovnjaki iz prakse, saj bo le s takšnim sodelovanjem mogoče soustvarjati podporno zdravstveno pismeno okolje v Sloveniji.

## 1 INTRODUCTION

Health literacy is gaining increasing attention in both scientific and political contexts, as it is associated with improved health outcomes, enabling people to effectively navigate complex healthcare systems, make informed decisions, adopt healthier behaviours, and enhance overall wellbeing, while also contributing to reducing health inequalities (1,2,3).

### 1.1 Health literacy research development

From a global perspective, the volume of scientific research in healthcare is increasing, as bibliometric analyses show a steady rise in publications and citations over time, reflecting the growing interest in and advancement of the field (4). A similar trend can be seen in health literacy.

A search for “health literacy” in PubMed, the largest online bibliographic database for biomedicine and healthcare, shows that from 1974, when the concept was first introduced (5), to 2000, only 758 publications mentioning health literacy were indexed.

In contrast, over the past 25 years, the number of publications has increased markedly. Between 2000 and 2025, 29,426 publications on this topic were indexed in the database. The number of publications has risen sharply over the past decade, reaching a peak in 2025 (4,314 articles).

But why? This growth is likely to reflect a range of factors. These include the modernisation of healthcare systems, with a greater focus on patient empowerment and person-centred care; the expansion of the Internet, digitalisation, and the increasing use of social media and digital platforms; the infodemic; and related developments (6,7,8,9,10).

### 1.2 Meeting the needs in a complex world

According to Sørensen et al. (11) “health literacy is linked to literacy and entails people's knowledge, motivation and competences to access, understand, appraise, and apply health information in order to make judgments and take decisions in everyday life concerning healthcare, disease prevention and health promotion to maintain or improve quality of life during the life course” (11). This definition of general health literacy reflects its relational and multidimensional nature, highlighting the interaction between personal skills and the demands contextual demands. It may also be analysed through its individual dimensions and domains to capture the full complexity of how people manage their health and wellbeing in different contexts.

Owing to the increasing complexity of the modern world and diverse needs, health literacy is content- and context-specific, according to Nutbeam. In his paper, he describes how functional, interactive, and critical health literacy influences how we understand, interact and critically appraise information to make decisions (12).

Other examples of how health literacy is being specified and contextualised include digital health literacy, vaccination literacy, and mental health literacy as exemplified in the 2023 editorial in Zdravstveno varstvo/Slovenian Journal of Public Health Kamin (13).

To mention just a few more, communicative health literacy refers to the communicative and social skills that enable people to actively engage in face-to-face encounters with healthcare professionals, to seek and provide information, to derive meaning from that information, and to apply it in decision-making processes and in the co-production of their healthcare (14).

Navigational health literacy is a specific form of health literacy that relates to the use of, and navigation within, the healthcare system (15), which is complex and fragmented (16). It encompasses a person's knowledge, motivation, and skills to access, understand, appraise, and apply information needed to navigate health care systems and services efficiently (17, 18). The navigational health literacy is particularly critical in the Slovenian context, which we will discuss below.

Environmental health literacy refers to the knowledge and skills that enable people to understand and act on the effects of environmental factors on health. It includes an understanding of environmental hazards, risk indicators, and decision-making aimed at reducing the harmful effects of the environment on health (19). Risk literacy is defined as the ability to understand and use information about risks, including statistical and probabilistic reasoning, which is essential for informed decision-making in healthcare. It describes how knowledge about risks influences decisions and the management of health (20). We may also add nutrition literacy (21), critical health literacy (22), media health literacy (23) and others that will require greater attention in the future.

As mentioned, health literacy can be understood as the knowledge and skills that people develop over time through everyday experiences, social interactions, and intergenerational learning. These skills are influenced by organisational structures and resource availability, which determine how effectively people can access, understand, evaluate, and use health information and services to support and maintain their own health and wellbeing, as well as that of others (24).

Lastly, attention should also be given to organisational health literacy, which refers to how well healthcare organisations support people in finding, understanding, and using information and services to make informed decisions about their health (25).

It is important to consider health literacy, including its various forms and dimensions, from both global and national perspectives. Particular attention should be paid to its implementation within national contexts, as cultural differences, healthcare systems, and other systemic factors, as well as social disparities and the needs of vulnerable populations, must be carefully taken into account.

## 2 GLOBAL PERSPECTIVES ON HEALTH LITERACY DEVELOPMENT

Beyond the high dissemination of health literacy research in Europe and North America, the pace and form of health literacy development differ considerably across settings in many countries in Africa, Asia and South America. Health literacy is closely intertwined with broader agendas on universal health coverage, primary health care, noncommunicable diseases, and digital health (26,27,28). This supports the gradual shift from viewing health literacy purely as an individual skill to understanding it as a relational and systemic capacity, shaped by language, culture, education systems, and the organisation of health services (29).

To address health literacy effectively, it is necessary to move beyond its growing research base and integrate it into governance. In recent years, global health literacy efforts have been reinforced by emerging policy frameworks and human rights–based approaches (30). A recent WHO integrative review of national health literacy policy blueprints shows how countries are using standalone strategies and action plans to build health-literate systems through intersectoral governance, capacity building, and systematic implementation at population and organisational levels (31). Moreover, the WHO has made health literacy a policy priority in its Global Health Strategy 2025–2028 to support resilience across member states.

In parallel, the Council of Europe's report, Health Literacy is a Human Rights Concern (32), frames health literacy as essential to realising the right to health, documenting multiple barriers for vulnerable groups (such as people on low incomes, those with lower levels of education, migrants and ethnic minorities, men, (33,34,35,36), children and adolescents (37) and calling for structural measures to reduce discrimination and improve equitable access to information, services, and care. Together, these developments position health literacy not only as a policy priority for effective, people-centred health systems but also as a requirement for upholding human dignity and health-related human rights across the life course.

Although there is globally growing interest in health literacy, some critics argue that this interest has not yet been translated into substantive advances in public health interventions (38), therefore, countries must incorporate health literacy into their strategic documents, which provide a framework for action, as well as into their operational action plans, through which they prioritise activities and define timelines for the implementation of concrete public health interventions through structured educational programmes, tailored communication strategies, and self-management support, particularly for people with chronic conditions and vulnerable populations. Such interventions have been shown to improve disease knowledge, treatment adherence, self-efficacy, and overall health outcomes, while also enhancing people's ability to navigate healthcare systems and reducing health inequalities (39,40,41,42).

## 3 SLOVENIAN PERSPECTIVE ON HEALTH LITERACY RESEARCH AND POLICY

To monitor and evaluate health literacy development and progress, population studies such as the European Health Literacy Survey, coordinated by the WHO Action Network on Measuring Population and Organisational Health Literacy, are being conducted in several European countries (43).

**Figure 1.**
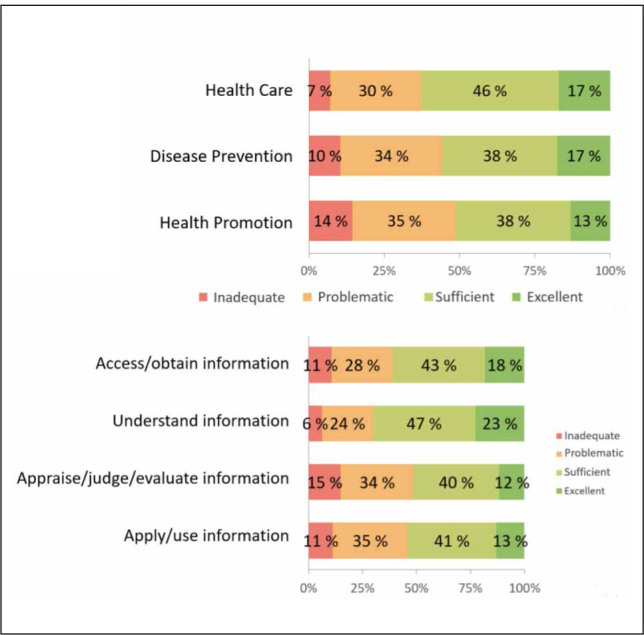
Categories of general health literacy achievements (n = 3.323–3.360), 2020.

To date, only one national study has examined health literacy among the adult population in Slovenia based on the M-POHL survey. Compared with other participating countries (bearing in mind differences in data collection methods and sampling procedures), Slovenia's results are encouraging (44). Nevertheless, 48% of the population in Slovenia were found to have limited health literacy (45). Differences were observed across individual domains and dimensions of health literacy, with the appraisal and application of health information emerging as particularly problematic dimensions (Figure 1).

Communicative health literacy among the population in Slovenia appears to be less problematic, as findings indicate that only 20% of adults have limited communicative health literacy. The main challenges were identified in areas such as having sufficient time during consultations, expressing personal opinions, and being actively involved in decisions about one's health when speaking with healthcare professionals (45).

In contrast, navigational health literacy presents a considerably greater challenge. As many as 61% of adults in Slovenia were found to have limited navigational health literacy. Particular difficulties were identified in understanding information about healthcare reforms that may affect access to care; assessing whether specific healthcare services meet their needs; understanding what is covered by health insurance; and finding information about patients' rights within the healthcare system (45). These findings underscore the importance of Slovenia addressing the challenges identified in this field.

### 3.1 The Slovenian National Health Literacy Strategy 2025–2035

A key step was the development of the National Health Literacy Strategy 2025–2035. The Ministry of Health invited a broad range of experts and stakeholders to collaborate on preparing the document, which the Government of the Republic of Slovenia subsequently adopted. The strategy aims to improve health literacy at both individual and organisational levels over the next decade.

The National Health Literacy Strategy of Slovenia 2025–2035 is the first comprehensive framework to address this area. It provides a basis for systematic, coordinated, and practical Public health interventions and programmes. Its primary objective is to improve the health literacy of the population, to be pursued through nine strategic sub-objectives: 1) Empowering the population of Slovenia by ensuring access to clear, comprehensible, reliable, and culturally appropriate health information; 2) Strengthening the role of healthcare organisations as health-literate organisations; 3) Developing the skills of healthcare professionals in health literacy; 4) Improving the health literacy of people with chronic diseases to promote empowerment, active participation, and improved self-management; 5) Promoting digital health literacy; 6) Improving population health literacy through a life-course approach, across diverse living environments, and with the involvement of civil society; 7) Advancing research and development in health literacy; 8) Advocating for and integrating health literacy into public policies and cross-sectoral collaboration; 9) Promoting international cooperation in health literacy.

Progress will be monitored using 111 measurable indicators (46). In addition to linking activities across key domains—particularly research and implementation—the strategy connects stakeholders and actions across sectors. It is based on the premise that effective progress requires simultaneous action at the individual and population levels, as well as within healthcare organisations and the broader system, to create supportive “health-literate environments”. Importantly, the strategy extends beyond the healthcare sector to include education and social services. The strategy is based on the concept that improving health literacy requires action at three interrelated levels: personal, professional, and organisational health literacy (47).

Health literacy may be operationalised into public health practice through systematically designed learning initiatives, context-sensitive and tailored communication approaches, and mechanisms that empower people to manage their own health, particularly among those living with chronic conditions and within vulnerable populations. A substantial body of evidence demonstrates that these efforts improve understanding of disease processes, promote consistent adherence to therapeutic regimens, reinforce self-efficacy, and ultimately result in more favourable health outcomes. Moreover, they strengthen people's ability to engage with and utilise healthcare services effectively, while also contributing to the mitigation of health disparities (48,49,50).

## 4 CONCLUSION

Continued research on health literacy is needed. Greater emphasis should be placed on interventional research and research on the development of health literacy. Data are essential; without them, conclusions are neither sound nor defensible. In 2026, Slovenia will conduct its second national health literacy survey among the adult population, providing insight into the current state of health literacy and offering a comparative perspective on health literacy levels prior to the COVID-19 pandemic. In addition to assessing population health literacy—ranging from general to specific forms—and identifying vulnerabilities, particular attention should be given to health literacy among children and adolescents. In Slovenia, no data are currently available for this population group, and significant research gaps remain internationally in this area.

Looking ahead, it will be important to create an environment in which decision-makers recognise health literacy as a key requirement for the effective delivery of health policies and wider public policy. This requires promoting an understanding of health literacy as a societal value or standard, rather than merely as an individual attribute.

Within the healthcare system, it is equally essential to foster awareness that higher levels of health literacy yield positive outcomes. Building health literacy, healthcare workers, and system capacity remains a call to action in Europe, and the new Slovenian health literacy strategy will help pave the way for stronger impact in the coming years.
